# Laplace approximation, penalized quasi-likelihood, and adaptive Gauss–Hermite quadrature for generalized linear mixed models: towards meta-analysis of binary outcome with sparse data

**DOI:** 10.1186/s12874-020-01035-6

**Published:** 2020-06-11

**Authors:** Ke Ju, Lifeng Lin, Haitao Chu, Liang-Liang Cheng, Chang Xu

**Affiliations:** 1 West China School of Public Health and West China Fourth Hospital, Sichuan University, Chengdu, China; 2 Department of Statistics, Florida State University, Tallahassee, FL USA; 3 Division of Biostatistics, School of Public Health, University of Minnesota, Minneapolis, MN USA; 4 School of Public Health, Sun Yat-sen University, Guangzhou, China; 5 Department of Population Medicine, College of Medicine, Qatar University, Al Jamiaa Street, P. O. Box 2713, Doha, Qatar

**Keywords:** Rare events, Meta-analysis, One-stage approach, Both-arm zero events

## Abstract

**Background:**

In meta-analyses of a binary outcome, double zero events in some studies cause a critical methodology problem. The generalized linear mixed model (GLMM) has been proposed as a valid statistical tool for pooling such data. Three parameter estimation methods, including the Laplace approximation (LA), penalized quasi-likelihood (PQL) and adaptive Gauss–Hermite quadrature (AGHQ) were frequently used in the GLMM. However, the performance of GLMM via these estimation methods is unclear in meta-analysis with zero events.

**Methods:**

A simulation study was conducted to compare the performance. We fitted five random-effects GLMMs and estimated the results through the LA, PQL and AGHQ methods, respectively. Each scenario conducted 20,000 simulation iterations. The data from Cochrane Database of Systematic Reviews were collected to form the simulation settings. The estimation methods were compared in terms of the convergence rate, bias, mean square error, and coverage probability.

**Results:**

Our results suggested that when the total events were insufficient in either of the arms, the GLMMs did not show good point estimation to pool studies of rare events. The AGHQ method did not show better properties than the LA estimation in terms of convergence rate, bias, coverage, and possibility to produce very large odds ratios. In addition, although the PQL had some advantages, it was not the preferred option due to its low convergence rate in some situations, and the suboptimal point and variance estimation compared to the LA.

**Conclusion:**

The GLMM is an alternative for meta-analysis of rare events and is especially useful in the presence of zero-events studies, while at least 10 total events in both arms is recommended when employing GLMM for meta-analysis. The penalized quasi-likelihood and adaptive Gauss–Hermite quadrature are not superior to the Laplace approximation for rare events and thus they are not recommended.

## Background

Meta-analysis is a statistical approach to synthesize the findings from similar studies to the same question and is widely used in healthcare science to make better decisions [1]. Classical meta-analytic methods are generally based on a two-stage framework (stage 1: forming the estimates from each original study; stage 2: pooling these estimates across studies), which assigns the effect sizes with a specific weighting scheme (e.g. inverse variance) and sums up the weighted effect sizes across the studies to achieve the goal of evidence pooling [2].

Dealing with zero events has been a critical problem in meta-analysis. When zero events occur in either of the arms, the effect size on a relative scale, e.g. odds ratio (OR), and its variance within the study are undefined, challenging the synthesis of such studies [3, 4]. Statisticians have since proposed several methods including the continuity correction, Mantel–Haenszel, and Yusuf–Peto as potential solutions, and these methods perform well under specific conditions (e.g. balanced sample size, one-arm zero events) [5–10]. However, when zero events occur in both arms, these methods have been proven to be questionable [6, 9].

In practice, researchers routinely discard trials that have zero events in both arms with the argument that such trials are non-informative for the treatment comparison. Unfortunately, this could be problematic for several reasons. As Kuss et al. claimed, “*both-zero studies with balanced sample size point to no differences in treatment effects and deleting them might bias the treatment effect … patients who have been recruited in double-zero studies have a right to their data being also included in meta-analyses*” [9]. Xie et al. have discussed this problem and advocated that zero-events studies contains inference information when assuming the underlying population events rate were not zero [10]. Based on the meta-analysis data from the Cochrane Database of Systematic Reviews, our investigation also verified that studies with no events in both arms contain information for inference [11].

The one-stage framework may serve as an alternative since it allows studies with no events to be contained for pooling [8, 12, 13]. As one of one-stage meta-analytic approaches, the generalized linear mixed model (GLMM), which treats individuals as level 1 and studies as level 2, is established to summarize the effect sizes directly within the multilevel regression model [8, 12–14]. Simmonds and Higgins have documented the general framework of GLMM for different types of meta-analysis [8]. Jackson et al. described six GLMMs for head-to-head comparison and compared them to the generic two-stage random-effects model and demonstrated that the GLMMs generally showed better statistical inference [15].

General linear models usually employ the maximum likelihood or restricted maximum likelihood method for parameter estimation. However, the GLMMs involve more complex random-effects variance components, so there is no closed form for the log likelihood, making the estimation intractable [16, 17]. Several methods were available as solutions to approximate the likelihood; they include the Laplace approximation (LA), the penalized quasi-likelihood (PQL) and the adaptive Gauss–Hermite quadrature (AGHQ) [17–19]. These methods are valid in certain situations and the AGHQ method has been regarded as the most accurate one among them [20]. For meta-analysis of rare events, there is currently no clear picture on the three methods’ performance. Thomas et al. have compared the performance of the PQL and AGHQ based on two standard GLMMs and demonstrated no meaningful difference between them [21]. However, the LA method and other GLMMs were not investigated in their simulation.

Jackson et al. and Thomas et al. [15, 21] made significant steps forward for the use of GLMMs on rare events and showed possibilities of solving the zero-events problem in meta-analysis. There are, however, two further questions that have not been well understood: 1) When GLMMs can be used for meta-analyses of rare events? 2) Do the PQL and AGHQ have better statistical properties than the LA in such meta-analyses? The elucidation of these two questions will have implications for methodological guidelines and evidence synthesis practice. This study reported the statistical properties of five random-effects GLMMs with the three parameter estimation methods (i.e. LA, PQL, AGHQ) by simulating meta-analyses of rare events. Some recommendations were also provided based on our findings.

## Methods

### The GLMMs

We consider five random-effects GLMM models described by Jackson et al. [15], including the random slope model (model 1), the random intercept and slope model (model 2), the modified random slope model (model 3), the modified random intercept and slope model (model 4), and the bivariate random slope model (model 5) [15]. All GLMMs are considered under a frequentist framework within this manuscript. Of note, these five methods are originally denoted as models 2 to 6 in Jackson et al. [15]. We consider these as random-effects models because all of them use a random slope in the GLMM framework. Let *i* index studies and *j* index treatment status (1 for treatment and 0 for control).

#### Model 1: the random slope model

The random slope model employs a random treatment effect term *θ*_*i*_~*N*(*θ*, *τ*^2^) with a fixed study effect (*γ*_*i*_) based on the multilevel logistic model. Here *θ*_*i*_ is the study-specific true log odds ratio. Denote the study-specific event rate by *π*_*ij*_, the model can be written as:

$$ \mathrm{logit}\left({\pi}_{ij}\right)={\gamma}_i+j{\theta}_i, $$

where *θ*_*i*_ = logit(*π*_*i*1_) − logit(*π*_*i*0_) = *θ* + *ε*_*i*_ and *ε*_*i*_ is the random error term with the variance of *τ*^2^, i.e. *ε*_*i*_~*N*(0, *τ*^2^). By expressing *θ*_*i*_ in terms of *θ* and *ε*_*i*_, the model can be written as:

$$ \mathrm{logit}\left({\pi}_{ij}\right)={\gamma}_i+ j\theta +j{\varepsilon}_i. $$

#### Model 2: the random intercept and slope model

The random intercept and slope model employs both a random study effect *γ*_*i*_~*N*(*γ*, *σ*^2^) and a random treatment effect *θ*_*i*_~*N*(*θ*, *τ*^2^); that is, this model considers both the between-study variance (*τ*^2^) and the variance of the study effect (baseline risk):

$$ \mathrm{logit}\left({\pi}_{ij}\right)={\gamma}_i+ j\theta +j{\varepsilon}_i. $$

#### Model 3: the modified random slope model

The modified random slope model is the modification of model 1 that uses a different parameterization by adding a design matrix with elements *z*_*ij*_*= j* − 0.5 for *θ*_*i*_ so that the elements in the variance of log odds for treatment effect are “averaged” (±[*τ*/2]^2^). Let us use the true effect *θ* and the random error *ε*_*i*_ to express *θ*_*i*_, i.e. *θ*_*i*_ = *θ* + *ε*_*i*_. Note that replacing *jθ* by *z*_*ij*_*θ* (0.5 for treatment and − 0.5 for control) does not change the form of the treatment status and is simply a model reparameterization. Consequently, this model can be written as:

$$ \mathrm{logit}\left({\pi}_{ij}\right)={\gamma}_i+{z}_{ij}\theta +{z}_{ij}{\varepsilon}_i. $$

#### Model 4: the modified random intercept and slope model

Model 4 is the modification of model 2 with respect to the variance-covariance structure. This model can also be derived from model 3 that assumes the study effect as random *γ*_*i*_~*N*(*γ*, *σ*^2^) [15]. Again, we use the true effect *θ* and the random error *ε*_*i*_ to express *θ*_*i*_, and model 4 is:

$$ \mathrm{logit}\left({\pi}_{ij}\right)={\gamma}_i+{z}_{ij}\theta +{z}_{ij}{\varepsilon}_i $$

It is notable that model 4 and model 3 have the same equation form, the distinguish could be that model 4 assumes study effect as random effect while model 3 as fixed effect.

#### Model 5: the modified bivariate random slope model

Unlike the previous four models, model 5 considers the potential correlation of the probability for an event of the two comparative arms from model 3. Therefore, it uses a bivariate GLMM structure with the slopes being random effects:

$$ \left[\begin{array}{c} logit\left({\pi}_{i0}\right)\\ {} logit\left({\pi}_{i1}\right)\end{array}\right]\sim N\left(\left(\begin{array}{c}\gamma \kern1.5em \\ {}\gamma +\theta \end{array}\right),\Sigma \right);\Sigma \sim \left(\begin{array}{cc}{\sigma}_0^2& {\rho \sigma}_0{\sigma}_1\\ {}{\rho \sigma}_0{\sigma}_1& {\sigma}_1^2\end{array}\right), $$

where ${\sigma}_1^2$ and ${\sigma}_0^2$ are the variances according to the event rates in treatment and control arms, respectively, and *ρ* is the correlation coefficient between them. Thus, the variance of *θ* is:

$$ {\tau}^2={\sigma}_0^2+{\sigma}_1^2-2\rho {\sigma}_0{\sigma}_1. $$

### The three parameter estimation methods

**Fig. 1:**
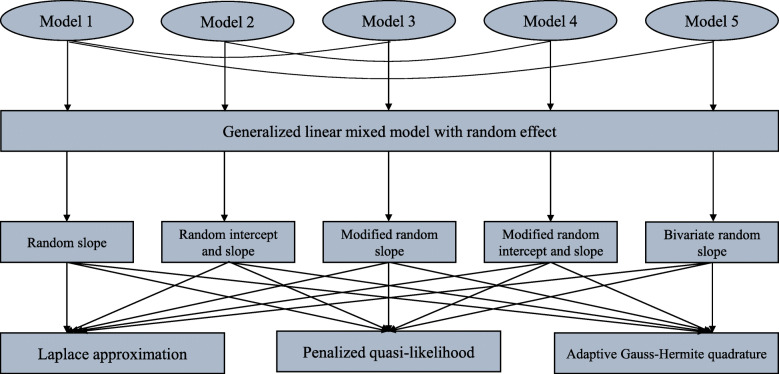
The five random-effects GLMM meta-analytic models

We consider three parameter estimation methods, i.e. the LA, PQL, and AGHQ methods; they were used for each GLMM. As a result, they led to a total of 15 (5 × 3) one-stage meta-analytic methods (Fig. 1). The LA method uses Taylor series expansion of the log-likelihood function and takes the first three terms (the second term is zero) to approximate the log-likelihood for a numerical solution. The PQL method uses the second-order approximation for the Taylor series expansion of the quasi-likelihood function to approximate the quasi-likelihood and obtain a solution. The AGHQ method uses the *n*^th^ power (here we use the default value *n* = 7) to minimax approximation for the log-likelihood function, with an adaptive procedure to refine the knots in order to reach a better approximation. A detailed description of the three methods has been documented elsewhere [17, 18].

**Fig. 2:**
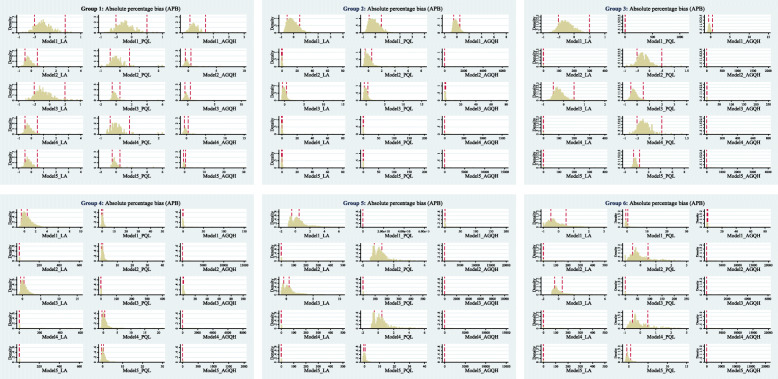
The performance of each GLMM model under different estimation method when the OR = 1 & Tau (*τ*) = 0.2

### Data generation

We used the “*pCFixed*” data-generating model for current simulation (grouped data) [22]. The empirical distribution of the meta-analysis data from the Cochrane Database of Systematic Reviews was used for the simulation [23, 24]. We identified 550 meta-analyses (with 4122 trials) that contained studies with no events [11], and the sample size information was fitted into 71 commonly-used distributions to estimate the optimal values for the parameters (sample size distribution) for simulating meta-analyses (by minimizing the sum of square errors). Based on the above, the log-normal distribution fitted well in both the treatment (*mean* = 3.4418, *standard error* = 0.9823) and the control (*mean* = 3.3537, *standard error* = 0.9992) arms of the sample size. Considering the potential correlations on the sample size of the two arms, we further analyzed the sample size ratio of them and utilized the ratio and the log-normal distribution of the control arm to get the sample size of the treatment arm. More specifically, a uniform distribution was fitted and then the first and the third quartiles of the sample size ratio were taken from the empirical Cochrane data (0.84–2.04). Let’s denote *n*_1_ and *n*_2_ as the sample size of treatment and control arms, then: log *n*_2_ ~ *N* (3.3537, 0.9992), *n*_1_ = exp. (log *n*_2_)*ratio, where *ratio* ~ uniform (0.84, 2.04).

The mean event risk in the control arm from the 4122 trials was 0.07; we however set it as 0.01 to improve the possibility for generating studies with zero events. This definition of rare events was also used in Jackson et al. [15]. For the true effect size, i.e. the odds ratio (OR), we considered five equally-spaced values from 1 to 5 and each log OR was normally distributed with the variance of *τ*^2^ across studies (i.e. between-study variance). For example, log OR ~ *N* (log (2), *τ*^2^). For the between-study variance, five monotonic *τ* from mild to substantial (0.2, 0.4, 0.6, 0.8, 1.0) were considered. The event risk in the treatment arm was then calculated by the risk in the control arm, the OR, and the between-study variance. We set the number of included studies as a uniform distribution ranged from 4 to 10 (step width: 1) for each meta-analysis based on the first and third quartiles of the empirical data [11]. Finally, a total of 25 (5 × 5) scenarios were considered according to the above conditions (Table 1).

**Table 1:** Simulation parameter setup

Parameter	Assigned values
Incidence rate of the control group (*pc*)	0.01
Number of patients in control group (*n*_2_)	mean (log) =3.3537, sd (log) =0.9992
Sample size ratio (*ratio*)	Uniform (0.84, 2.04)
Number of patients in experimental group (*n*_1_)	*n*_1_ = exp. (log *n*_2_)*ratio
Effect sizes (*OR*)	1, 2, 3, 4, 5
Between-study variance (*τ*^2^)	*τ*=(0.2, 0.4, 0.6, 0.8, 1.0)
Number of studies included in each meta-analysis (*m*)	Uniform (4, 10)

### Data analysis

The following measures were used to assess each model’s performance:

- Convergence rate, defined as the ratio of the number of iterations that generated finite estimates over the total number after excluding zero-event meta-analyses;
- Percentage bias (PB), calculated as: PB = (*OR* − *OR*_True_)/*OR*_True_ × 100%;
- Mean squared error (MSE), calculated as: MSE = Var(*OR*) + (*OR* − *OR*_True_)^2^;
- Coverage probability, i.e. the probability of the 95% CI containing the true value among every 1000 iterations;

The PB reflects the unbiasedness of a point estimate (e.g. regression coefficient) with a lower value indicating smaller bias. The MSE measures both the point and variance estimation. The coverage reflects the ability to cover the true value. Theoretically, a coverage of 95% under 95% confidence level is optimal. Considering that PB and MSE are not normally distributed (with long tails), we compared their medians instead of their mean values. For such types of distribution, the median value is usually smaller than the mean value. For the PB, we pre-defined the acceptable percentage as 50%; the proportion of meta-analyses exceeding this cutoff point was reported as the primary index to rank the properties of the models.

To better understand the statistical properties for the LA, PQL, and AGHQ, we stratified the number of events for treatment and control arms in each meta-analysis by the following total events setting scheme:

Group 1: Both arms ≥10;

Group 2: One arm ≥10 and one ≥5 but less than 10;

Group 3: Both arms ≥5 but less than 10;

Group 4: One arm > = 10 and one < 5.

Group 5: One arm ≥5 while < 10 and another arm < 5;

Group 6: Both arms less than 5.

This was because a previous simulation study for logistic regression suggested that 10 events for each variable would be stable for the estimation, 5–10 would be somewhat stable, and less than 5 would be unstable [25]. We excluded those with zero total events in its treatment and/or control arm, because none of the three methods was valid in this situation by producing infinite estimates.

We simulated 20,000 iterations (meta-analyses) for each scenario. All simulations and analyses were conducted using the R software (version 3.4.2) with the “lme4” and “GLMMadaptive” packages [26, 27]. The Stata14.0/SE (STATA, College Station, TX) and Excel 2013 (Microsoft, America) were used for visualization of the results. The R code for meta-analysis is provided in the Additional file 1.

## Results

### LA, PQL, and AGHQ

#### Convergence rate

Table 2 presents the convergence rate of the three estimation methods. The LA kept consistently high convergence rate (100% in most of the cases) in all models. The AGHQ method kept a similar high convergence rate in Models 1 and 3 while the rate reduced by about 5–10% in Models 2, 4, and 5 when OR = 1. For the PQL method, Models 2, 4, and 5 had extremely low convergence rates (< 20% in most of the cases). Models 1 and 3 based on the PQL kept a high convergence rate similar to the LA and AGHQ. The results enlightened that, due to the low convergence rate, the PQL estimation procedure was not the optimal option when fitting models with two random-effects terms or bivariate term.

**Table 2:** Convergence rate for each estimation procedure in each scenario (based on 20,000 iterations)

Procedure	Convergence rate (0–100%)																								
	OR = 1					OR = 2					OR = 3					OR = 4					OR = 5				
	*τ*=0.2	*τ*=0.4	*τ*==0.6	*τ*=0.8	*τ*=1.0	*τ*=0.2	*τ*=0.4	*τ*==0.6	*τ*=0.8	*τ*=1.0	*τ*=0.2	*τ*=0.4	*τ*==0.6	*τ*=0.8	*τ*=1.0	*τ*=0.2	*τ*=0.4	*τ*==0.6	*τ*=0.8	*τ*=1.0	*τ*=0.2	*τ*=0.4	*τ*==0.6	*τ*=0.8	*τ*=1.0
**Laplace Approximation (LA)**																									
Model 1	100	100	100	100	100	100	100	100	100	100	100	100	100	100	100	100	100	100	100	100	100	100	100	100	100
Model 2	100	100	100	100	100	100	100	100	100	100	100	100	100	100	100	100	100	100	100	100	100	100	100	100	100
Model 3	100	100	100	100	100	100	100	100	100	100	100	100	100	99.99	100	100	100	100	99.99	100	100	100	100	100	100
Model 4	100	100	100	100	100	100	100	100	100	100	100	100	100	99.99	100	100	99.99	100	99.99	100	100	100	100	100	100
Model 5	100	100	100	100	100	100	100	100	100	100	100	100	100	100	100	100	99.99	100	100	100	100	100	100	100	100
**Penalized quasi-likelihood estimation (PQL)**																									
Model 1	98.46	98.47	98.25	97.48	96.31	99.07	99.01	98.94	98.51	97.01	99.08	99.14	99.01	98.52	96.30	99.27	99.38	99.17	98.58	93.22	99.50	99.44	99.39	98.84	96.84
Model 2	16.37	19.01	17.23	16.16	13.51	17.03	16.97	16.48	15.20	13.76	17.45	17.07	17.08	15.66	14.04	18.85	18.41	17.64	15.84	14.28	19.65	19.62	18.51	16.25	14.56
Model 3	99.99	99.98	99.99	99.96	99.95	99.99	100	99.99	99.99	99.93	99.92	99.92	99.91	99.86	99.86	99.89	99.93	99.93	99.84	99.42	100	99.99	100	99.98	99.95
Model 4	15.59	18.50	16.13	13.06	9.91	16.24	15.82	15.23	12.55	9.49	16.97	16.48	15.01	12.49	8.43	17.94	17.58	15.48	12.21	6.32	18.63	18.05	15.80	11.55	7.82
Model 5	19.67	22.22	19.52	16.57	14.08	20.57	20.15	19.24	16.77	13.72	21.31	20.73	19.22	16.74	13.14	22.73	22.04	20.25	16.31	11.41	23.96	23.16	20.37	15.94	12.50
**Adaptive Gauss-Hermite quadrature (AGHQ)**																									
Model 1	98.87	99.14	99.01	98.98	98.12	99.97	99.98	99.67	99.97	99.97	99.43	99.47	99.24	99.16	98.64	99.67	99.53	99.50	98.96	98.87	99.99	99.99	99.99	99.98	99.98
Model 2	86.59	89.45	88.34	87.69	81.55	99.50	99.47	99.41	99.31	99.22	96.26	96.15	95.13	94.70	92.77	96.40	96.60	95.87	94.44	92.44	99.63	99.64	99.70	99.60	99.49
Model 3	99.07	99.32	99.01	98.69	97.62	99.97	99.96	99.95	99.93	99.98	99.49	99.47	99.10	98.59	97.66	99.57	99.58	99.34	98.72	98.02	99.98	99.96	99.96	99.96	99.98
Model 4	87.09	89.15	88.06	87.44	82.10	98.72	98.64	98.35	97.85	97.26	96.07	95.97	95.45	94.24	92.58	96.74	96.51	96.49	94.65	92.79	99.29	99.24	98.85	97.93	97.31
Model 5	90.96	92.11	91.35	89.90	86.51	99.73	99.73	99.71	99.65	99.69	96.58	96.64	96.12	94.96	93.77	97.20	97.12	96.08	94.50	91.17	99.79	99.74	99.72	99.70	99.60

#### Bias

Figure 2 (OR = 1, *τ* =0.2) shows the distribution of the PB of the three estimation methods under different total events settings. A small proportion of them had very large bias based on the LA and PQL methods, while a large proportion of very large bias occurred on the AGHQ method. We did not plot the distribution graph for other scenarios (e.g. OR = 2, *τ* =0.4), because as the between-study variance increased there would be huge bias that impacted the visualization

#### Large ORs

Very large bias occurred when ORs were very large. This is due to the systematic error when the total events are rare. Table 3 (OR = 1, *τ* =0.2) and Table S1 (All scenarios) summarize the proportion of large ORs (defined as OR ≥ 250 [27]) for different estimation methods. Under the PQL method, a large proportion (> 80%) of large ORs occurred in Model 2, 4, and 5, while a low proportion in Model 1 and 3.

**Table 3:** The proportion of large ORs in each estimation procedure under different models

Models	Model 1	Model 2	Model 3	Model 4	Model 5
**OR = 1 (***τ***=0.2)**					
LA	0.22%	0.32%	0.00%	0.29%	0.3%
PQL	1.88%	83.74%	0.09%	84.41%	80.5%
AGHQ	1.25%	14.79%	1.17%	14.39%	10.22%
**OR = 2 (***τ***=0.2)**					
LA	0.03%	0.43%	0.00%	0.43%	0.43%
PQL	1.03%	83.01%	0.15%	83.76%	79.63%
AGHQ	0.03%	0.50%	0.03%	1.28%	0.27%
**OR = 3 (***τ***=0.2)**					
LA	0.00%	0.68%	0.00%	0.66%	0.70%
PQL	0.96%	82.55%	0.31%	83.03%	79.01%
AGHQ	0.72%	4.98%	0.69%	5.39%	4.52%
**OR = 4 (***τ***=0.2)**					
LA	0.00%	1.00%	0.00%	0.94%	1.02%
PQL	0.74%	81.15%	0.41%	82.06%	77.49%
AGHQ	0.48%	4.90%	0.59%	4.78%	4.07%
**OR = 5 (***τ***=0.2)**					
LA	0.00%	0.74%	0.00%	0.74%	0.77%
PQL	0.50%	80.35%	0.21%	81.38%	76.24%
AGHQ	0.01%	0.37%	0.02%	0.71%	0.21%

All the results were based on 20,000 iterations

Under the LA method, a low proportion (less than 1.02%) that produced large ORs was observed, regardless of which models utilized. The AGHQ method had a low, but slightly higher proportion of large ORs than the LA method.

#### Sectional summary

- The PQL and AGHQ did not show better properties than the LA in terms of convergence rate, bias, and probability to generate large ORs.

### Total events and GLMM properties

The number of meta-analyses in each group is shown in Table S2. Generally, the number of meta-analyses in each group was sufficient. Group 4 had the largest number of meta-analyses. It is notable that, in two scenarios (OR = 4 and 5), the number of meta-analyses in Group 3 were small. Therefore, in this section, we did not use the data of OR = 4 and 5 for comparison to avoid the large uncertainty due to the small number of observations [28].

**Fig. 3:**
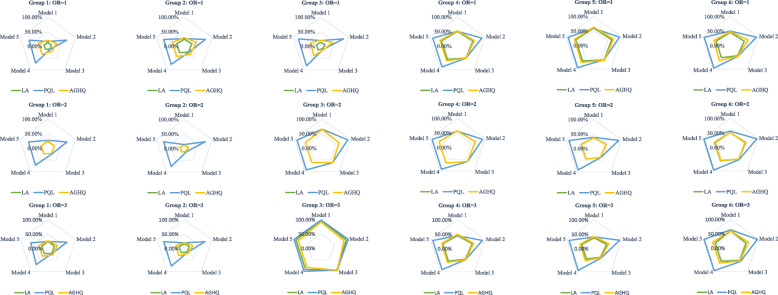
The proportion of percentage bias larger than 50% under different models and estimation methods when the Tau (*τ*) = 0.2

Figure 3 shows the performance (*τ* =0.2). From Group 3 to Group 6, most meta-analyses had biases larger than 50%, regardless of the estimation method utilized. In Group 1 and Group 2 the biases were much smaller. We observed that when OR = 1, Group 1 had lower proportion of bias that larger than 50% compared to Group 2; while when OR > 1, inverted results occurred. This is because when the events in two arms were comparable (Group 1), the pooled OR converged to 1, and thus in Group 1 the proportion of bias > 50% was lower when OR = 1; otherwise, when the events in two arms were incomparable (Group 2), the pooled OR diverged to 1, and thus in Group 1 the proportion of bias > 50% was higher when OR > 1.

Again, the PQL estimation had a poor performance based on Model 2, 4, and 5, even if the total events were relative sufficient (Group 1 and 2). The LA estimation had lower proportions of bias larger than 50% than the AGHQ in all of the situations. The results were similar in other scenarios (e.g. *τ* =0.4), but they became worse as the between-study variance increased (Figures S1–S4). As a consequence, when the total events were insufficient (Group 3–6) in either of the arms, the GLMMs did not show good point estimation to pool studies of rare events.

#### Sectional summary

- When the total events were insufficient in either of the arms, the GLMMs did not show good point estimation to pool studies of rare events. Conservatively, at least 10 total events in both arms were needed when employing GLMMs for meta-analysis.

### Five random-effects GLMMs

Accounting for the above findings, we compared the performance of remaining potential models, including all five random-effects models (Models 1 to 5) based on LA estimation, all five random-effect models based on AGHQ, and the two classical models (Models 1 and 3) based on the PQL. Therefore, 12 models were further compared in total.

#### Bias

**Fig. 4:**
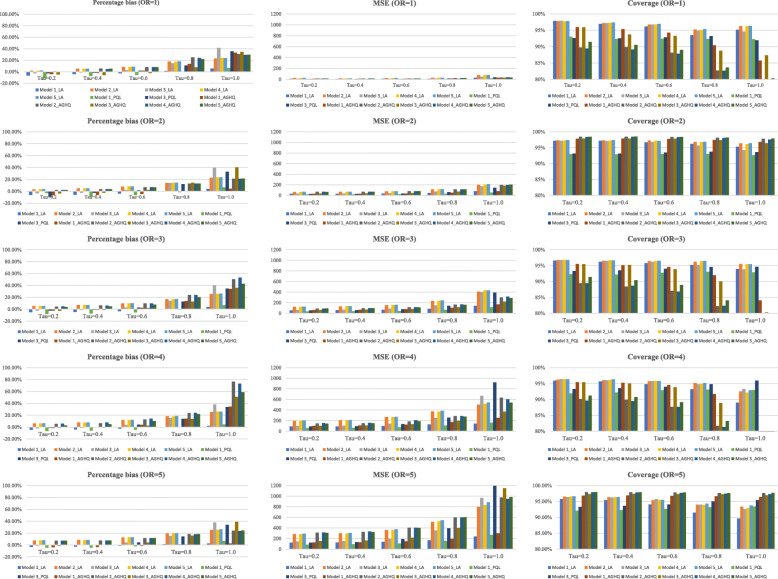
The comparison of the median percentage bias, MSE, and coverage probability for the 12 models

Figure 4 compares the 12 models in terms of the median PB. Generally, Model 1 had a low bias regardless of which estimation method was utilized. Model 3 had lower bias than Model 1 when the between-study variance was not large (*τ* <=0.6). However, when there was large variance (*τ* > = 0.8), Model 3 had larger bias. Model 2, 4, and 5 had similar amount of bias (lower than Model 1 when OR < 3 while larger when OR > 3), and the bias in Model 5 was slightly larger. These three were less susceptible to the changes of between-study variance than Model 3. On average, the PB of GLMMs ranged from − 11.24% to 13.05 (less than 10% on absolute scale in most of the cases) when the between-study variance was not large (*τ* <=0.6); as the variance increased the bias also increased, but less than 40% in most of the cases.

#### Mean squared error

Again, Model 1 and Model 3 had the lowest MSE in most of the cases and Model 3 generally had higher MSE than Model 1. But for Model 3 based on PQL estimation, the MSE were large when the between-study variance was large (*τ* =1.0). Model 1 based on the PQL estimation (green bar) had the lowest MSE and Model 1 based on the LA estimation (blue bar) had the second lowest MSE. Model 2, 4, and 5 have similar MSEs that larger than Model 1 and 3. A larger between-study variance and/or a larger effect size generally led to a larger MSE.

#### Coverage

As expected, Model 2, 4 and 5 generally had better coverage than Model 1 and 3. We observed that different estimation procedures (i.e. LA, PQL, AGHQ) had some impacts on the coverage probability: models based on LA tend to have better coverage than models based on AGHQ and PQL. The two models based on the PQL had coverage probabilities under the normal level (95%) in almost all cases. In 14 out of the 25 scenarios, Model 2, 4, and 5 based on AGHQ estimation had coverage probabilities under the normal level. The five random-effects models based on the LA had good coverage probabilities in most situations (*τ* <= 0.6). As the between-study variance increased, the coverage decreased, especially for Model 1 and 3.

#### Sectional summary

- The AGHQ and PQL were not superior to the LA with respect to bias, MSE, and coverage.
- Model 1 and 3 had lower bias and MSE than Model 2, 4, and 5, while the later three had better coverage and were less susceptible to variance on bias;
- When there was large between-study variance, none of these models had a good performance.

## Discussion

In this study, we compared the statistical properties of five random-effects GLMMs and three parameter estimation methods (LA, PQL, AGHQ) by simulations for meta-analyses of rare events. Based on the findings, when the total events were insufficient (e.g. less than 10) in either of the arms, the GLMMs did not show good point estimation to pool studies of rare events. The AGHQ estimation method did not show better properties than the LA estimation. We further found that although the PQL had some advantages, it was neither the preferred option due to the low convergence rate in some situations nor the suboptimal point and variance estimation.

We observed that the Model 2, 4, and 5 had some advantages in dealing with heterogeneous studies (i.e. less susceptible to between-study variance on bias and better coverage), which has been described in Jackson et al. [15]. This could be expected – by fitting with two random-effects terms or a bivariate term, they give more “freedom” to estimate the difference [13]. And this is why the MSE tend to be larger than the random slope models (Model 1 and 3). These properties allow the above models to generate a more conservative estimation. However, the bias of them tends to be large when compared to the random slope models (Model 1 and 3). We further found that, although Model 1 and Model 3 performed better in light of bias and MSE but showed lower coverage possibility. This suggested that some caution should be noticed as the results were at risk of overconfident by Model 1 and 3 when the between-study variance was large.

In our simulation, there was no evidence that the PQL and AGHQ showed better statistical properties even when the total events were rare. The PQL had a low convergence rate when modeled with Model 2, 4, and Model 5. This is because these three models involve more parameters to be estimated than Model 1 and 3. Our results suggested that when the total events were insufficient (Group 3–6), none of the three estimation methods performs well. The Firth’s logistic regression based on the penalized maximum likelihood is a potential solution for it [29]. However, it is infeasible to establish a multilevel model for Firth’s logistic regression, and no software package is currently available for its implementation. Whether the penalized maximum likelihood faces the same problem (less convergent) in random-effect models is unclear. A further investigation on multilevel firth’s regression on meta-analysis of rare events would be valuable for this topic.

Based on the pros and cons of these models and the simulation results, we propose some recommendations for model selection. First, studies with no events in both arms contain information for inference and GLMMs can serve as a valid method to pool such studies [11]. Second, when using GLMMs to pool studies with rare events, meta-analysts should ensure a sufficient number of total events in both arms (i.e., ≥10). Third, we do not suggest to use GLMMs with the PQL or AGHQ estimation method; the LA has sufficiently satisfactory performance. Fourth, when there is substantial variance between studies, the bias increases significantly that the results should be treated with caution.

This study’s strength includes that we used empirical data to determine the simulation settings, so our comparisons and results were closer to reality. We investigated the applicability of the GLMMs for meta-analysis of rare events and verified the least requirement on total events. We also verified that the PQL or AGHQ estimation did not show better properties than the LA estimation. To the best of our knowledge, this is the first simulation study that address these questions. Our study is expected to provide potential guidance for further systematic reviews and meta-analyses. Several limitations should be highlighted. The first one would be the number of simulations. Although there were 20,000 iterations for each scenario, we noticed that in some groups (e.g. group 3) the number of iterations were relatively small. And the limited observations may hamper the credibility the between-group comparisons. The second one is the data-generation mechanism that was applied. This mechanism assumes that all the heterogeneity in the simulation is placed on the treatment arm. This simulative strategy introduces unequal number of zero events between the experimental and the control group, which possibly has implications in the comparisons as well (see [22] for details).

## Conclusion

The GLMM is an alternative for meta-analysis of rare events and is especially useful in the presence of no-events studies; however, this model should be used with caution when the total events are insufficient. Conservatively, at least 10 total events in both arms were needed when employing GLMM for meta-analysis. The penalized quasi-likelihood and adaptive Gauss–Hermite quadrature are not superior to the Laplace approximation for rare events and usually take much longer computing time thus they are not recommended.

## Supplementary information

**Additional file 1: Table S1.** The proportion of large ORs in each estimation procedure under different models. **Table S2.** Number of simulated meta-analyses in each group. **Figure S1.** The proportion of which with bias larger than 50% under different models and estimation methods when the Tau = 0.4. **Figure S2.** The proportion of which with bias larger than 50% under different models and estimation methods when the Tau = 0.6. **Figure S3.** The proportion of which with bias larger than 50% under different models and estimation methods when the Tau = 0.8. **Figure S4.** The proportion of which with bias larger than 50% under different models and estimation methods when the Tau = 1.0.

## Data Availability

Data can be obtained by contacting the corresponding author.

## Abbreviations

GLMM: Generalized linear mixed model
LA: Laplace approximation
PQL: Penalized quasi-likelihood
AGHQ: Adaptive Gauss–Hermite quadrature
PB: Percentage bias
MSE: Mean squared error
OR: Odds ratio
CI: Confidence interval
