# Supplementary material for: Laplace approximation, penalized quasi-likelihood, and adaptive Gauss–Hermite quadrature for generalized linear mixed models: towards meta-analysis of binary outcome with sparse data
Source: BMC Med Res Methodol. 2020 Jun 11;20:152. doi: 10.1186/s12874-020-01035-6 (PMC7296731; doi:10.1186/s12874-020-01035-6)

Laplace approximation, penalized quasi-likelihood, and adaptive Gauss-Hermite quadrature for generalized linear mixed models: Towards meta-analysis of binary outcome with sparse data

Ke Ju^1^, Lifeng Lin^2^, Haitao Chu^3^, Liang-Liang Cheng^4^, Chang Xu^5🖂^

^1^ West China School of Public Health, Sichuan University, Chengdu, China;

^2^ Department of Statistics, Florida State University, Tallahassee, FL, USA;

^3^ Division of Biostatistics, School of Public Health, University of Minnesota, Minneapolis, MN, USA;

^4^ School of Public Health, Sun Yat-sen University, Guangzhou, China;

^5^ Department of Population Medicine, College of Medicine, Qatar University, Doha, Qatar;

**Running title:** Generalized linear mixed models for meta-analysis of sparse data

## ^🖂^Corresponding to:

Dr. Chang Xu, Department of Population Medicine, College of Medicine, Qatar University, Al Jamiaa Street, P. O. Box 2713, Doha, Qatar, Email: [xuchang2016@runbox.com](mailto:xuchang2016@runbox.com).

## R code used in the meta-analysis

**1.1 Indexes**

*Study* denotes the studies in a meta-analysis; *n* denotes the total number; *event* denotes the number of events; *Treat* involves 1/0, and denotes treatment group and control group, respectively; *Treat12* involves 0.5/-0.5, which is gained by Treat minus 1; *Control* involves 0/1, which is contrary to Treat.

**1.2 R code for 15 models**

*Model1_LA <- glmer(cbind(event,(n-event)) ~ factor(treat) + ((treat-1)|study), data = meta_data, family = binomial(link = "logit"))*

*Model1_PQL <- glmmPQL(cbind(event,(n-event)) ~ factor(treat),random = ~(treat-1) | study, family = binomial(link="logit"), data = meta_data)*

*Model1_AGHQ <- mixed_model(fixed = cbind(event,(n-event)) ~ factor(treat) , random = ~(treat-1) | study,family = binomial(link="logit"), data = meta_data)*

*Model2_ LA <- glmer(cbind(event,(n-event)) ~ factor(treat) + (treat|study), data = meta_data, family = binomial(link = "logit"))*

*Model2_PQL <- glmmPQL(cbind(event,(n-event)) ~ factor(treat) ,random = ~ treat | study,family = binomial(link="logit"), data = meta_data)*

*Model2_AGHQ <- mixed_model(fixed = cbind(event,(n-event)) ~ factor(treat) , random = ~treat | study,family = binomial(link="logit"), data = meta_data)*

*Model3_ LA <- glmer(cbind(event,(n-event)) ~ factor(treat) + ((treat12-1)|study), data = meta_data, family = binomial(link = "logit"))*

*Model3_PQL <- glmmPQL(cbind(event,(n-event)) ~ factor(treat) ,random = ~ (treat12-1) | study,family = binomial(link="logit"), data = meta_data)*

*Model3_AGHQ <- mixed_model(fixed = cbind(event,(n-event)) ~ factor(treat) , random = ~(treat12-1) | study,family = binomial(link="logit"), data = meta_data)*

*Model4_ LA <- glmer(cbind(event,(n-event)) ~ factor(treat) + ((treat12)|study), data = meta_data, family = binomial(link = "logit"))*

*Model4_PQL <- glmmPQL(cbind(event,(n-event)) ~ factor(treat) ,random = ~ treat12 | study,family = binomial(link="logit"), data = meta_data)*

*Model4_AGHQ <- mixed_model(fixed = cbind(event,(n-event)) ~ factor(treat) , random = ~treat12 | study,family = binomial(link="logit"), data = meta_data)*

*Model5_ LA <- glmer(cbind(event,(n-event)) ~ factor(treat) + ((control+treat-1)|study), data = meta_data, family = binomial(link = "logit"))*

*Model5_PQL <- glmmPQL(cbind(event,(n-event)) ~ factor(treat) ,random = ~ (control+treat-1) | study,family = binomial(link="logit"), data = meta_data)*

*Model5_AGHQ <- mixed_model(fixed = cbind(event,(n-event)) ~ factor(treat) , random = ~(control+treat-1) | study,family = binomial(link="logit"), data = meta_data)*

**Table S1.** The proportion of large ORs in each estimation procedure under different models.

| **Models** | **Model 1** | | | | | **Model 2** | | | | | **Model 3** | | | | | **Model 4** | | | | | **Model 5** | | | | |
| --- | --- | --- | --- | --- | --- | --- | --- | --- | --- | --- | --- | --- | --- | --- | --- | --- | --- | --- | --- | --- | --- | --- | --- | --- | --- |
| **Tau** | **Tau = 0.2** | **Tau = 0.4** | **Tau = 0.6** | **Tau = 0.8** | **Tau = 1.0** | **Tau = 0.2** | **Tau = 0.4** | **Tau = 0.6** | **Tau = 0.8** | **Tau = 1.0** | **Tau = 0.2** | **Tau = 0.4** | **Tau = 0.6** | **Tau = 0.8** | **Tau = 1.0** | **Tau = 0.2** | **Tau = 0.4** | **Tau = 0.6** | **Tau = 0.8** | **Tau = 1.0** | **Tau = 0.2** | **Tau = 0.4** | **Tau = 0.6** | **Tau = 0.8** | **Tau = 1.0** |
| **OR=1** | | | | | |  | | | | |  | | | | |  | | | | |  | | | | |
| LA | 0.22% | 0.12% | 0.15% | 0.12% | 0.78% | 0.32% | 0.30% | 0.32% | 0.38% | 0.70% | 0.00% | 0.00% | 0.00% | 0.00% | 0.00% | 0.29% | 0.25% | 0.30% | 0.30% | 0.66% | 0.3% | 0.30% | 0.31% | 0.34% | 0.68% |
| PQL | 1.88% | 1.75% | 1.97% | 2.77% | 4.67% | 83.74% | 81.06% | 82.86% | 83.94% | 86.79% | 0.09% | 0.09% | 0.12% | 0.22% | 0.30% | 84.41% | 81.50% | 83.87% | 86.95% | 90.10% | 80.5% | 77.97% | 80.66% | 83.59% | 86.11% |
| AGHQ | 1.25% | 0.92% | 1.05% | 1.13% | 2.10% | 14.79% | 11.72% | 12.81% | 13.35% | 20.23% | 1.17% | 0.91% | 1.22% | 1.58% | 3.16% | 14.39% | 11.96% | 13.12% | 13.87% | 20.10% | 10.22% | 8.90% | 9.71% | 11.37% | 15.71% |
| **OR=2** | | | | | |  | | | | |  | | | | |  | | | | |  | | | | |
| LA | 0.03% | 0.06% | 0.06% | 0.11% | 0.25% | 0.43% | 0.43% | 0.39% | 0.44% | 0.54% | 0.00% | 0.00% | 0.00% | 0.00% | 0.00% | 0.43% | 0.43% | 0.37% | 0.39% | 0.50% | 0.43% | 0.43% | 0.38% | 0.43% | 0.52% |
| PQL | 1.03% | 1.10% | 1.23% | 1.79% | 3.62% | 83.01% | 83.09% | 83.57% | 84.88% | 86.43% | 0.15% | 0.15% | 0.09% | 0.15% | 0.40% | 83.76% | 84.18% | 84.77% | 87.45% | 90.51% | 79.63% | 80.04% | 80.92% | 83.37% | 86.47% |
| AGHQ | 0.03% | 0.02% | 0.03% | 0.03% | 0.03% | 0.50% | 0.53% | 0.59% | 0.71% | 0.85% | 0.03% | 0.04% | 0.05% | 0.07% | 0.02% | 1.28% | 1.36% | 1.65% | 2.16% | 2.79% | 0.27% | 0.28% | 0.29% | 0.38% | 0.41% |
| **OR=3** | | | | | |  | | | | |  | | | | |  | | | | |  | | | | |
| LA | 0.00% | 0.00% | 0.00% | 0.06% | 0.22% | 0.68% | 0.71% | 0.69% | 0.64% | 0.86% | 0.00% | 0.00% | 0.00% | 0.01% | 0.00% | 0.66% | 0.72% | 0.68% | 0.64% | 0.85% | 0.70% | 0.73% | 0.69% | 0.63% | 0.89% |
| PQL | 0.96% | 0.93% | 1.06% | 1.64% | 4.18% | 82.55% | 82.93% | 82.92% | 84.40% | 86.17% | 0.31% | 0.31% | 0.41% | 0.53% | 0.76% | 83.03% | 83.52% | 84.99% | 87.51% | 91.57% | 79.01% | 79.54% | 81.02% | 83.54% | 87.07% |
| AGHQ | 0.72% | 0.72% | 0.95% | 1.23% | 2.03% | 4.98% | 5.32% | 6.29% | 7.27% | 10.24% | 0.69% | 0.76% | 1.26% | 2.07% | 4.13% | 5.39% | 5.65% | 6.20% | 7.76% | 10.55% | 4.52% | 4.52% | 5.15% | 6.95% | 9.77% |
| **OR=4** | | | | | |  | | | | |  | | | | |  | | | | |  | | | | |
| LA | 0.00% | 0.00% | 0.00% | 0.03% | 0.03% | 1.00% | 1.02% | 0.82% | 0.82% | 1.98% | 0.00% | 0.00% | 0.00% | 0.01% | 0.01% | 0.94% | 0.94% | 0.82% | 0.81% | 1.85% | 1.02% | 1.03% | 0.84% | 0.80% | 2.10% |
| PQL | 0.74% | 0.63% | 0.73% | 1.45% | 7.24% | 81.15% | 81.59% | 82.36% | 84.16% | 85.90% | 0.41% | 0.43% | 0.53% | 0.70% | 1.29% | 82.06% | 82.42% | 84.52% | 87.79% | 93.68% | 77.49% | 78.17% | 80.10% | 84.01% | 88.91% |
| AGHQ | 0.48% | 0.70% | 0.68% | 1.32% | 2.05% | 4.90% | 4.71% | 5.69% | 7.79% | 11.30% | 0.59% | 0.60% | 0.97% | 2.10% | 3.82% | 4.78% | 4.83% | 5.42% | 7.54% | 11.03% | 4.07% | 4.03% | 5.41% | 7.61% | 12.84% |
| **OR=5** | | | | | |  | | | | |  | | | | |  | | | | |  | | | | |
| LA | 0.00% | 0.00% | 0.00% | 0.01% | 0.05% | 0.74% | 0.76% | 0.74% | 0.81% | 1.13% | 0.00% | 0.00% | 0.00% | 0.00% | 0.00% | 0.74% | 0.72% | 0.72% | 0.80% | 1.13% | 0.77% | 0.78% | 0.75% | 0.82% | 1.15% |
| PQL | 0.50% | 0.56% | 0.62% | 1.18% | 3.35% | 80.35% | 80.38% | 81.49% | 83.76% | 85.51% | 0.21% | 0.21% | 0.30% | 0.44% | 0.68% | 81.38% | 81.95% | 84.20% | 88.45% | 92.18% | 76.24% | 77.09% | 79.82% | 84.28% | 87.68% |
| AGHQ | 0.01% | 0.01% | 0.01% | 0.02% | 0.02% | 0.37% | 0.36% | 0.30% | 0.46% | 0.83% | 0.02% | 0.04% | 0.04% | 0.04% | 0.02% | 0.71% | 0.76% | 1.16% | 2.14% | 3.03% | 0.21% | 0.30% | 0.31% | 0.42% | 0.84% |

**Table S2.** Number of simulated meta-analyses in each group.

| **Groups** | **OR=1** | | | | | **OR=2** | | | | | **OR=3** | | | | | **OR=4** | | | | | **OR=5** | | | | |
| --- | --- | --- | --- | --- | --- | --- | --- | --- | --- | --- | --- | --- | --- | --- | --- | --- | --- | --- | --- | --- | --- | --- | --- | --- | --- |
|  | Tau=0.2 | Tau=0.4 | Tau=0.6 | Tau=0.8 | Tau=1.0 | Tau=0.2 | Tau=0.4 | Tau=0.6 | Tau=0.8 | Tau=1.0 | Tau=0.2 | Tau=0.4 | Tau=0.6 | Tau=0.8 | Tau=1.0 | Tau=0.2 | Tau=0.4 | Tau=0.6 | Tau=0.8 | Tau=1.0 | Tau=0.2 | Tau=0.4 | Tau=0.6 | Tau=0.8 | Tau=1.0 |
| **Group 1** | 352 | 454 | 460 | 500 | 399 | 545 | 543 | 543 | 537 | 531 | 476 | 503 | 496 | 497 | 476 | 505 | 497 | 498 | 495 | 574 | 560 | 560 | 560 | 560 | 560 |
| **Group 2** | 1,918 | 3,045 | 3,069 | 3,945 | 2,605 | 3,403 | 3,435 | 3,527 | 3,634 | 3,809 | 4,250 | 4,200 | 4,214 | 4,192 | 4,214 | 4,430 | 4,457 | 4,440 | 4,502 | 4,620 | 4,759 | 4,756 | 4,748 | 4,734 | 4,714 |
| **Group 3** | 1,858 | 1,078 | 1,075 | 570 | 1,464 | 1,230 | 1,178 | 1,095 | 989 | 819 | 341 | 339 | 300 | 309 | 304 | 112 | 117 | 132 | 138 | 65 | 51 | 54 | 61 | 74 | 88 |
| **Group 4** | 2,837 | 6,175 | 6,284 | 8,787 | 4,016 | 4,273 | 4,369 | 4,688 | 5,527 | 6,825 | 7,574 | 7,708 | 7,873 | 8,442 | 9,144 | 9,849 | 9,839 | 9,897 | 10,102 | 11,855 | 11,153 | 11,127 | 11,165 | 11,218 | 11,313 |
| **Group 5** | 4,766 | 3,402 | 3,434 | 2,345 | 4,695 | 5,852 | 5,791 | 5,553 | 4,928 | 3,991 | 4,084 | 4,042 | 3,948 | 3,457 | 2,816 | 2,583 | 2,519 | 2,464 | 2,228 | 707 | 1,408 | 1,444 | 1,406 | 1,339 | 1,185 |
| **Group 6** | 5,915 | 3,693 | 3,520 | 1,908 | 4,499 | 2,769 | 2,754 | 2,662 | 2,454 | 2,067 | 1,255 | 1,220 | 1,175 | 1,114 | 1,032 | 541 | 550 | 545 | 513 | 221 | 216 | 206 | 207 | 219 | 282 |
| **Total** | 17,646 | 17,847 | 17,842 | 18,055 | 17,678 | 18,072 | 18,070 | 18,068 | 18,069 | 18,042 | 17,980 | 18,012 | 18,006 | 18,011 | 17,986 | 18,020 | 17,979 | 17,976 | 17,978 | 18,042 | 18,147 | 18,147 | 18,147 | 18,144 | 18,142 |

**Figure S1.** The proportion of which with bias larger than 50% under different models and estimation methods when the Tau = 0.4.


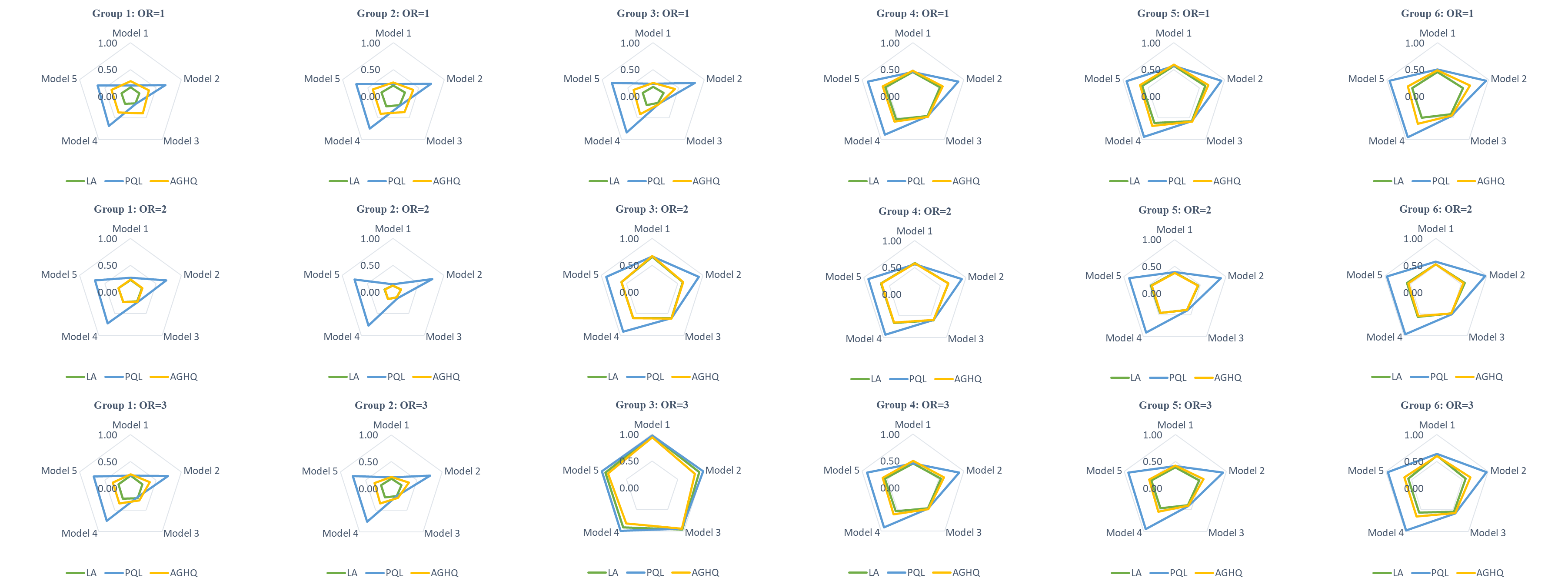


**Figure S2.** The proportion of which with bias larger than 50% under different models and estimation methods when the Tau = 0.6.


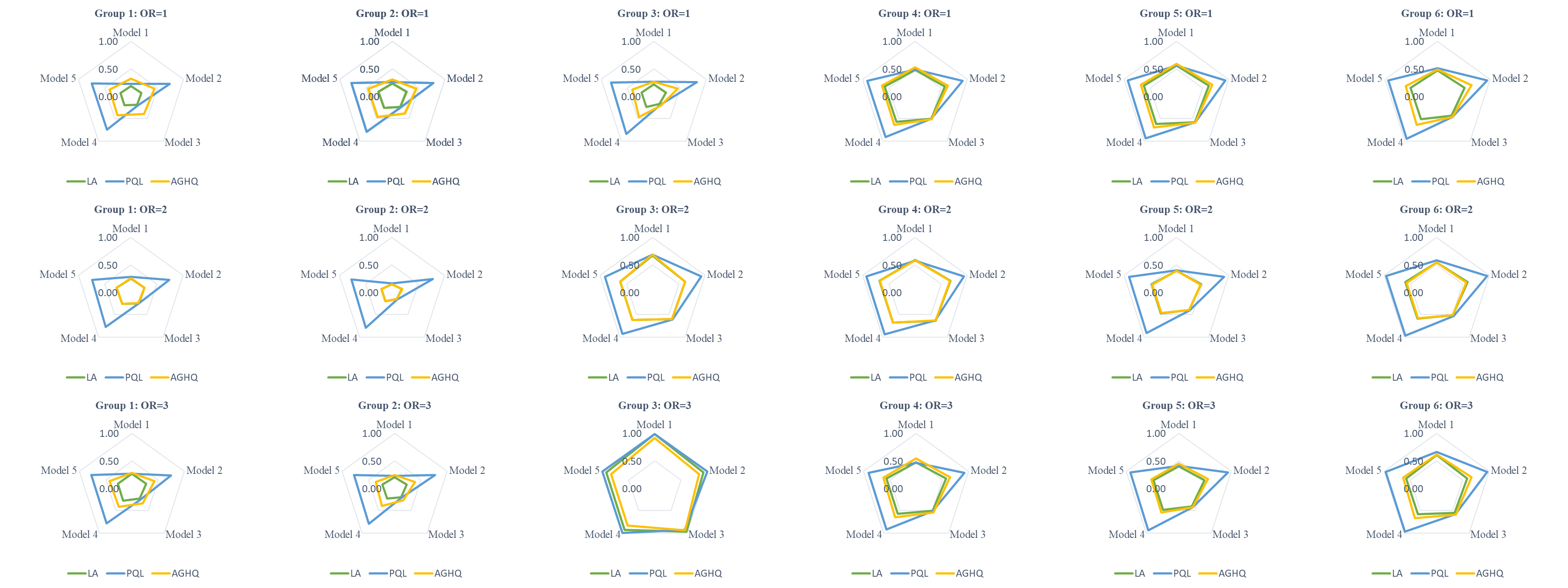


**Figure S3.** The proportion of which with bias larger than 50% under different models and estimation methods when the Tau = 0.8.


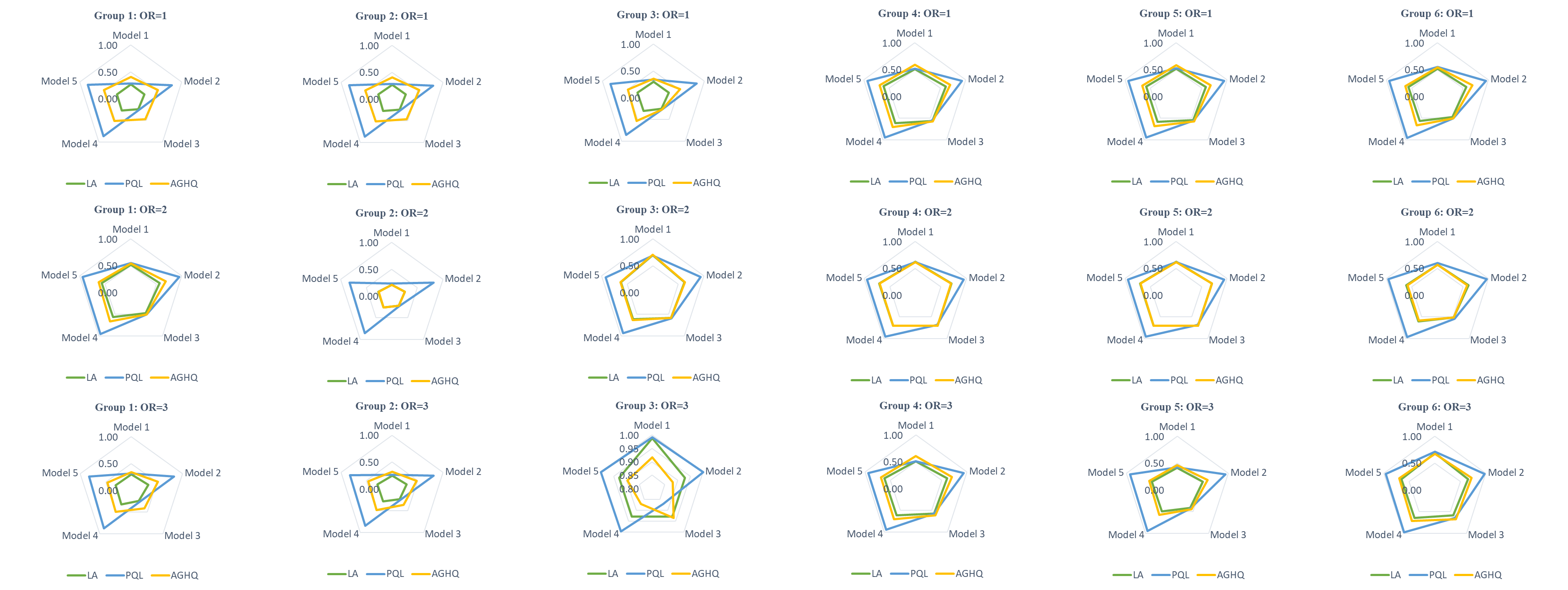


**Figure S4.** The proportion of which with bias larger than 50% under different models and estimation methods when the Tau = 1.0.


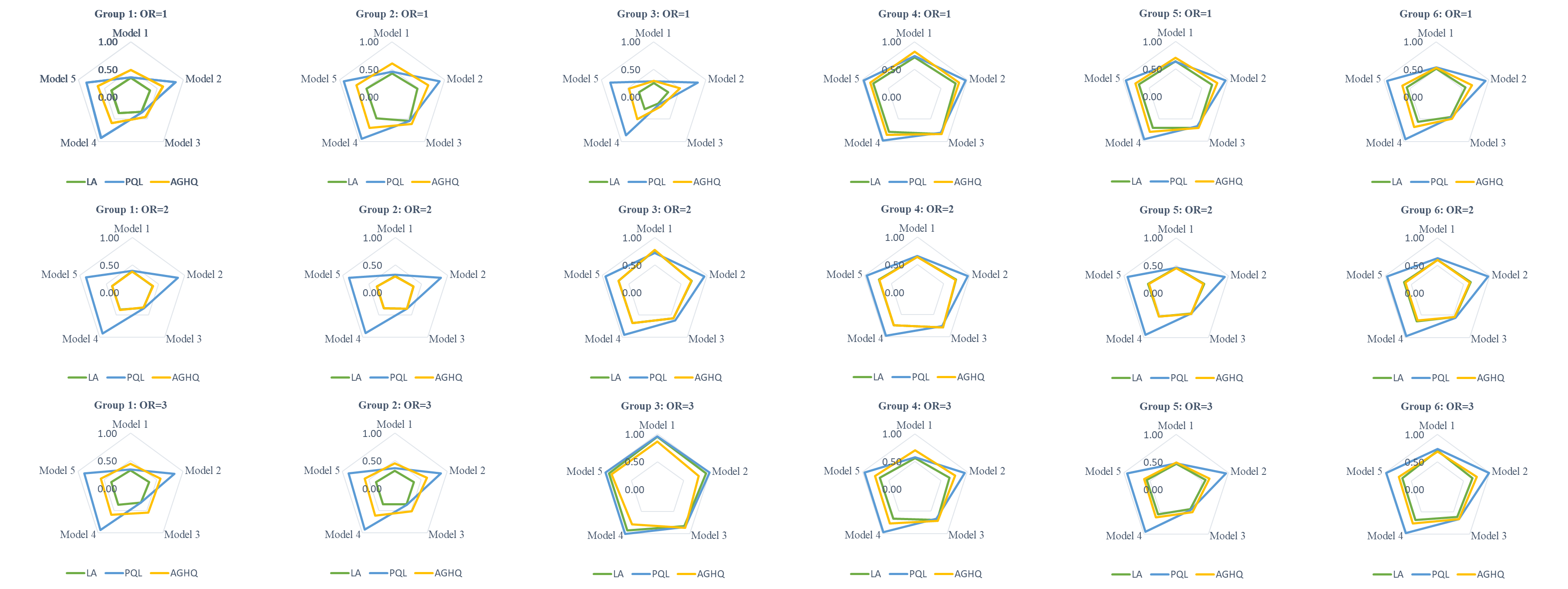

Supplement: Supplementary file 1 — Additional file 1: Table S1. The proportion of large ORs in each estimation procedure under different models. Table S2. Number of simulated meta-analyses in each group. Figure S1. The proportion of which with bias larger than 50% under different models and estimation methods when the Tau = 0.4. Figure S2. The proportion of which with bias larger than 50% under different models and estimation methods when the Tau = 0.6. Figure S3. The proportion of which with bias larger than 50% under different models and estimation methods when the Tau = 0.8. Figure S4. The proportion of which with bias larger than 50% under different models and estimation methods when the Tau = 1.0. [file 12874_2020_1035_MOESM1_ESM.docx]
